# Supplementary material for: DGCyTOF: Deep learning with graphic cluster visualization to predict cell types of single cell mass cytometry data
Source: PLoS Comput Biol. 2022 Apr 11;18(4):e1008885. doi: 10.1371/journal.pcbi.1008885 (PMC9060369; doi:10.1371/journal.pcbi.1008885)
Supplement: Attachment — Submitted filename: PCOMPBIOL-D-21-00432_comments_response.docx [file pcbi.1008885.s001.docx]

**Manuscript ID:** PLOS Computational Biology (PCOMPBIOL-D-21-00432) - [EMID:f6482cf2aa5d5c9f]

**Type of manuscript:** Article

**Title**: DGCyTOF:  deep learning with graphic cluster visualization to predict cell types of single cell mass cytometry data

**Authors**: Lijun Cheng , Pratik Karkhanis, Birkan Gokbag, and Lang Li

We thank the reviewer for his/her generous comments on the manuscript and we have edited the manuscript to address those concerns.

We would like to thank the reviewers for their constructive feedbacks and helpful suggestions. We have updated the manuscript. All revising is highlighted in yellow in paper. The following were updated:

1) we added a new comparison with and without dimension reduction and updated the Tables 4 – 5.

2) we updated GitHub API and associated coding.

The following information is the response to each of the reviewer comments:

**Reviewer #1**:

In the manuscript titled 'DGCyTOF: deep learning with graphic cluster visualization to predict cell types of single-cell mass cytometry data", the authors created a new pipeline to classify single cells from CyTOF data into known cell types. The pipeline also allows users to identify clusters that cannot fit into known cell types.

While some ideas of the pipeline are novel, such as the calibration step to refine to cell populations, I have many concerns about the proposed method.

**Comment 1.1**:

The authors showed the performance of the classification using two datasets. However, in each experiment, the reported performance is based on training and validation data spit from the same dataset. **How will the model perform in the presence of batch effects?** The whole purpose of deep-learning is to learn a generalizable model from one labeled dataset and apply the model to classify data from future experiments/studies.

**Response**:

Thank you for your feedback. DGCyTOF utilizes calibration on the same experiment samples for assigning new cell types to their respective groups. In this study, our proposed model run CCA (Canonical Correlation Analysis) method to remove the batch effect prior to using this model.

**Comment 1.2**:

An important use of automated clustering is to identify new cell subsets within known cell types. For example, there may be multiple cell subsets within CD8+ T cells, and their proportions could be associated with a disease. Why only limiting the cell clustering to cells with unknown label?

**Response**:

Thank you for your suggestion. Our model currently focuses on identifying cell populations given the supplied cell labels, working only on the classification tasks. The main goal was to assess the performance of novel calibration method on the cell identification task prior to extending it on other tasks. Identifying cell subsets from known cell labels and identifying differentiation across same cell type groups can be addressed in a future study.

**Comment 1.3**:

While UMAP performs the best when preserving the data's local structure, there will still be information loss during dimension reduction. It is not clear to me why the authors did not choose to **perform clustering using original data**. Can the author add another column in table 4 and 5 showing clustering performance without dimensionality reduction?

**Response**:

Thank you for your suggestion. we added a new comparison with and without dimension reduction to observe the loss of dimension reduction. An interesting result is that the performance of data dimensionality reduction+clustering is much better than clustering directly without dimension reduction. The results illustrate that dimensionality reduction contributes to main signal extraction during clustering or classification (Tables 4-5). We have updated the tables 4 and 5 with an additional column for no dimensionality reduction paired with the clustering methods as the follows.

**Table 4.** Comparison of machine-learning methods by different measurements for CyTOF Dataset 1 (13 biomarkers, 24 labeled cell types)

| Methods | | Measurement | *PCA* | *FA* | *ICA* | *Isomap* | *UMAP*  (DGCyTOF) | *t*-SNE |  | *NO-reduction* |
| --- | --- | --- | --- | --- | --- | --- | --- | --- | --- | --- |
| Dimension- reduction method | | Computation time  (in *seconds*) ↓ | 0.017 | 3.618 | 0.060 | 0.020 | 18.806 | 94.466 |  |  |
|  |  | *NPE* **↓** | 0.626 | 0.633 | 0.625 | 0.626 | 0.462 | 0.423 |  |  |
|  |  | *Retained variance* **↓** | 40.6% | 33.4% | - | 40.61% | N/A | N/A |  |  |
|  |  | Visualization **↑** | DD | DD | DD | DD | DD | ED |  |  |
| *k*-means clustering | | *F-score* **↑** | 0.286 | 0.282 | 0.288 | 0.269 | 0.565 | 0.507 |  | 0.286 |
|  |  | *ARI* **↑** | 0.236 | 0.228 | 0.236 | 0.216 | 0.556 | 0.483 |  | 0.235 |
|  |  | *FMI* **↑** | 0.307 | 0.298 | 0.307 | 0.286 | 0.627 | 0.563 |  | 0.306 |
|  |  | *V-measure* ↑ | 0.494 | 0.488 | 0.495 | 0.477 | 0.793 | 0.762 |  | 0.494 |
| Gaussian  mixture model | | *F-score* ↑ | 0.305 | 0.304 | 0.505 | 0.285 | 0.588 | 0.502 |  | 0.530 |
|  |  | *ARI* ↑ | 0.247 | 0.258 | 0.436 | 0.229 | 0.538 | 0.493 |  | 0.494 |
|  |  | *FMI* ↑ | 0.317 | 0.326 | 0.544 | 0.298 | 0.608 | 0.573 |  | 0.556 |
|  |  | *V-measure* ↑ | 0.497 | 0.490 | 0.586 | 0.481 | 0.790 | 0.768 |  | 0.704 |
| *HDBSCAN*  (DGCyTOF) | | *F-score* ↑ | 0.442 | 0.451 | 0.442 | 0.438 | **0.924** | 0.771 |  | 0.596 |
|  |  | *ARI* ↑ | 0.336 | 0.349 | 0.337 | 0.332 | **0.915** | 0.738 |  | 0.534 |
|  |  | *FMI* ↑ | 0.526 | 0.531 | 0.524 | 0.524 | **0.925** | 0.789 |  | 0.621 |
|  |  | *V-measure* **↑** | 0.557 | 0.570 | 0.552 | 0.555 | **0.905** | 0.850 |  | 0.557 |
|  | **Note** – All arrow indicators showed good trends. ARI, adjusted Rand index, measure of similarity between two clusters, involves random labeling independent of the number of clusters; DD, difficult to distinguish; ED, easy to distinguish; *FMI*, Fowlkes-Mallows score, geometric mean of pair-wise precision and recall; *F-score*, harmonic mean of precision and recall (values range from 0 [bad] to 1 [good]); *NPE*, neighborhood proportion error; *V-measure*, harmonic mean of homogeneity and completeness. All results reflect comparison of two dimensions, and the number of nearest neighbors (k) is 20. | | | | | | | | | |

**Table 5.** Comparison of machine-learning methods by different measurements for CyTOF Dataset 2 (32 biomarkers, 14 labeled cell types)

| Methods | Measurement | *PCA* | *FA* | *ICA* | *Isomap* | *UMAP*  (DGCyTOF) | *t-SNE* | *NO-reduction* |
| --- | --- | --- | --- | --- | --- | --- | --- | --- |
| Dimension- reduction method | Computation time  (in *seconds*) **↓** | 0.0253 | 0.208 | 0.054 | 0.021 | 16.944 | 95.609 |  |
|  | *NPE* **↓** | 0.536 | 0.525 | 0.535 | 0.536 | 0.399 | 0.393 |  |
|  | *Retained variance* **↓** | 31.03% | 27.70% | - | 31.03% | N/A | N/A |  |
|  | Visualization **↑** | DD | DD | DD | DD | **ED** | ED |  |
| K-means clustering | *F-score* **↑** | 0.426 | 0.421 | 0.431 | 0.322 | 0.590 | 0.529 | 0.458 |
|  | *ARI* **↑** | 0.343 | 0.330 | 0.349 | 0.232 | 0.540 | 0.475 | 0.409 |
|  | *FMI* **↑** | 0.444 | 0.431 | 0.448 | 0.340 | 0.637 | 0.578 | 0.537 |
|  | *V-measure* ↑ | 0.609 | 0.582 | 0.611 | 0.444 | 0.799 | 0.744 | 0.735 |
| Gaussian  mixture model | *F-score* ↑ | 0.497 | 0.446 | 0.670 | 0.313 | 0.626 | 0.585 | 0.395 |
|  | *ARI* ↑ | 0.406 | 0.353 | 0.573 | 0.221 | 0.577 | 0.534 | 0.339 |
|  | *FMI* ↑ | 0.500 | 0.453 | 0.706 | 0.331 | 0.665 | 0.631 | 0.461 |
|  | *V-measure* ↑ | 0.636 | 0.589 | 0.690 | 0.443 | 0.807 | 0.785 | 0.684 |
| *HDBSCAN*  (DGCyTOF) | *F-score* ↑ | 0.669 | 0.650 | 0.665 | 0.560 | **0.981** | 0.923 | 0.684 |
|  | *ARI* ↑ | 0.573 | 0.547 | 0.569 | 0.417 | **0.977** | 0.907 | 0.601 |
|  | *FMI* ↑ | 0.696 | 0.680 | 0.691 | 0.616 | **0.981** | 0.923 | 0.696 |
|  | *V-measure* **↑** | 0.698 | 0.657 | 0.691 | 0.565 | **0.952** | 0.898 | 0.701 |
| Note – All arrow indicators showed good trends. ARI, adjusted Rand index, measure of similarity between two clusters, involves random labeling independent of the number of clusters; DD, difficult to distinguish; ED, easy to distinguish; *FMI*, Fowlkes-Mallows score, geometric mean of pair-wise precision and recall; *F-score*, harmonic mean of precision and recall (values range from 0 [bad] to 1 [good]); *NPE*, neighborhood proportion error; *V-measure*, harmonic mean of homogeneity and completeness. All results reflect comparison of two dimensions, and the number of nearest neighbors (k) is 20. | | | | | | | | |

**Comment 1.4**:

The pipeline includes several hyper-parameters, including the number of layers in the DL model, the number of nodes in each layer, the threshold for softmax probability, and the correlation threshold for calibration (not to mention the hyperparameters in HDBSCAN and UMAP). How are the hyperparameters chosen? Is the test dataset used for hyper-parameter selection?

**Response**:

Thank you for your feedback. Default parameters were chosen for umap and tsne.

For hdbscan, min_samples was set to 1 to have the least conservative clustering, preventing data points from registering as noise in the clusters. min_cluster_size parameter was set to 10 to allow unique clusters to appear and prevent significant clusters from overshadowing newly found/unidentified clusters.

We used weight regularization to reduce overfitting of Deep Learning (DL) models. Our deep learning model’s hyperparameter tuning was done utilizing a regularization $l_{1}$ normalization to penalize weights sparse to 0 and selected the top 5% parameters [1]. On the other hand, we designed the dropout and dense strategy for parameters selection to prevent neural networks from overfitting [2-3]. The test dataset is not used for hyper-parameter selection, only for calibration step.

**Comment 1.5**:

The authors shared the package in Github. However, I did not see any documentation other than the installation guide. A concise vignette will be helpful for users to get started.

**Response**:

Thank you for your feedback.

The project’s website is located in <https://lijcheng12.github.io/DGCyTOF/> as description in the paper. Please click the button (view on GitHub) above the picture, the following is a screen shot . All documents list there.

The first page in GitHub, we list the API for function explanation and sample code are located in

<https://github.com/lijcheng12/DGCyTOF/blob/main/DGCyTOF_Package/docs/API.md>.

**Comment 1.6**:

The authors mentioned that "However, relying on clustering is laborious since it often involves manual annotation, which significantly limits the reproducibility of identifying cell-populations across different samples." It is unclear how the proposed clustering method (UMAP + HDBSCAN) improves the reproducibility of identifying cell populations **across different samples**. Is the clustering stable if applied to individual samples?

**Response**:

Thank you for your question. UMAP + HDBSCAN improves the reproducibility by utilizing hierarchy-density based clustering on the topologically created projection by the UMAP. This allows consistent results as the hierarchical density clustering integrates well with data that is already topologically addressed prior to use after batch effect removement adjusting. To across different samples, we will map all these samples in a low dimension space by UMAP and run CCA (Canonical Correlation Analysis) method to remove the batch effect, then look for the common clustering.

**Reviewer #2:**

The authors presented a deep learning framework, termed DGCyTOF, for embedding visualization of single-cell datasets. Specifically, DGCyTOF combines deep learning classification and hierarchical stable-clustering methods to sequentially build a tri-layer construct for known cell types and the identification of new cell types. Based on systematic evaluation, the authors showed a higher performance of the proposed DGCyTOF compared to Principal Component Analysis (PCA), Factor Analysis (FA), Independent Component Analysis (ICA), Isometric Feature Mapping (Isomap), t-distributed Stochastic Neighbor Embedding (t-SNE), and Uniform Manifold Approximation and Projection (UMAP) with k-means clustering and Gaussian mixture clustering. Overall, this is a novel study, which provides a powerful deep learning approach for single-cell data visualization. The manuscript is well-written and the overall computational evaluation is solid. Several specific minor comments should be considered further.

**Comment 2.1**:

One major concern is hyperparameter tuning for DGCyTOF and other comparing approaches, including PCA, UMAP, t-SNE, and others. The authors are suggested to provide the detailed process for hyperparameter tuning.

**Response**:

Thank you for your feedback. Default parameters were chosen for umap and tsne.

For hdbscan, min_samples was set to 1 to have the least conservative clustering, preventing data points from registering as noise in the clusters. min_cluster_size parameter was set to 10 to allow unique clusters to appear and prevent significant clusters from overshadowing newly found/unidentified clusters.

We used weight regularization to reduce overfitting of Deep Learning (DL) models. Our deep learning model’s hyperparameter tuning was done utilizing a regularization $l_{1}$ normalization to penalize weights sparse to 0 and selected the top 5% parameters [1]. On the other hand, we designed the dropout and dense strategy for parameters selection to prevent neural networks from overfitting [2-3]. The test dataset is not used for hyper-parameter selection, only for calibration step.

**Comment 2.2**:
As shown figure 3, the authors illustrated AUC =1 for most cell types. The authors are suggested to discuss possible risk of over-fitting.

**Response**:

Thank you for your feedback. We used 4-fold cross-validation between the true and prediction labels and overcome the overfitting. Our DGCyTOF result is the same as the model DeepCyTOF performance. The possible risk of overfitting in this deep learning model is based on how sparse the original data and features. To CyTOF datasets, deep learning model would work very well because the largest number of features is 50.

**Comment 2.3**:
The authors are suggested to discuss future studies about how to improve biological interpretation of deep learning models.

**Response**:

Thank you for your suggestion. For successful biological interpretation via deep learning models, it is vital that the results are reproducible and directly integrated to the biological phenomenon that is being analyzed. For future studies, the model can be extended to identify new cell type populations within already known cell type groups or be integrated with other databases as prior knowledge computing biological dependencies in the calculated result.

**Comment 2.4**:
Finally, computational cost and complexity of DGCyTOF should be compared with other approaches as well, such as PCA, UMAP, t-SNE, and others.

**Response**:

Thank you for your feedback. The computational cost and complexity of DGCyTOF (UMAP + HDBSCAN) is in the same category as the UMAP + HDBSCAN due to the method being an extension of the suggested dimensionality reduction technique and clustering algorithm. As deep learning is utilized, the actual time taken is dependent on the architecture the software was run on. In this study, the model was run on the OSC supercomputer with GPU integration for parallel processing.

**Reference**

[1] J.E. Moody. Note on generalization, regularization and architecture selection in nonlinear learning systems, Neural Networks for Signal Processing Proceedings of the 1991 IEEE Workshop.

[2] Behnam Neyshabur, Ryota Tomioka & Nathan Srebro. In search of the real inductive-bias on the role of implicit regularization in deep learning. 2015, The International Conference on Learning Representations (ICLR) 2015. arXiv:1412.6614.

[3] Nitish Srivastava, Geoffrey Hinton, Alex Krizhevsky, Ilya Sutskever, Ruslan Salakhutdinov; Dropout: A Simple Way to Prevent Neural Networks from Overfitting. 2014, Journal of machine learning research 15(56):1929−1958.
