# Supplementary material for: DGCyTOF: Deep learning with graphic cluster visualization to predict cell types of single cell mass cytometry data
Source: PLoS Comput Biol. 2022 Apr 11;18(4):e1008885. doi: 10.1371/journal.pcbi.1008885 (PMC9060369; doi:10.1371/journal.pcbi.1008885)
Supplement: Attachment — Submitted filename: PCOMPBIOL-D-21-00432R1_comments_response.docx [file pcbi.1008885.s002.docx]

**Manuscript ID:** PLOS Computational Biology (PCOMPBIOL-D-21-00432R1) - [EMID:71723ca9cdde4f8d]

**Type of manuscript:** Article

**Title**: DGCyTOF:  deep learning with graphic cluster visualization to predict cell types of single cell mass cytometry data

**Authors**: Lijun Cheng , Pratik Karkhanis, Birkan Gokbag, and Lang Li*

We thank the reviewer for his/her generous comments on the manuscript and we have edited the manuscript to address those concerns.

We would like to thank the reviewers for their constructive feedbacks and helpful suggestions. We have updated the manuscript. All revising is highlighted in yellow in paper.

The following information is the response to each of the reviewer comments:

**Reviewer #1**:

In the revised version of the manuscript titled "DGCyTOF: deep learning with graphic cluster visualization to predict cell types of single-cell mass cytometry data", the authors have addressed most of my concerns by adding new evaluation results and more detailed descriptions of the methods. However, two of my questions remain to be answered.

**Comment 1.1**:

First, how does DGCyTOF deal with the batch effect? In the point-by-point response, the author has mentioned using CCA (Canonical Correlation Analysis) to adjust for batch effect. However, I can't seem to find the description of CCA in the manuscript.

**Response**:

Thank you for your feedback. DGCyTOF utilizes calibration on the same experiment samples for assigning new cell types to their respective groups. In this study, Seurat MultiCCA method (Canonical Correlation Analysis) [[1](https://genomebiology.biomedcentral.com/articles/10.1186/s13059-019-1850-9#ref-CR4)] is used to remove the batch effect prior to use DGCyTOF, which was developed in 2017 by the Satija lab [[1](https://genomebiology.biomedcentral.com/articles/10.1186/s13059-019-1850-9#ref-CR4)]. Seurat uses CCA [[2](https://genomebiology.biomedcentral.com/articles/10.1186/s13059-019-1850-9#ref-CR11)] to project the data into a subspace to identify correlations across datasets to reduce data dimensionality and capture the most correlated data features to align the data batches.

**Comment 1.2**:

Second, it is still unclear to me how DGCyTOF is optimized. For example, how does the author determine the number of layers in the neural network and the number of nodes in each layer? The authors also mentioned using l1 regularization and drop-outs. How were the penalization weight (lambda) and the drop-out rates determined? Is there a cross-validation procedure for model optimization?

**Response**:

Thank you for your feedback. To classify cell types, we trained depth-N feed-forward neural nets, each consisting of three ***softplus*** hidden layers and a ***softmax*** output layer. To determine the optimal number of layers, penalization weight ($\lambda$) and their drop-out rate in the neural network, DGCyTOF utilized an automated pruning way of building neural network to improve computational and classification performance. By using particle swarm-optimized-based pruning algorithm [3], we selected the optimal multiple parameters (the number of layers, lambda, drop-out rate) in Python during network training. This objective maximization is to keep with the classify accuracy solution that maximizes the area under the *Receiver operating characteristic (ROC)* curve between real labels and prediction labels.

**Reviewer #2:**

The reviewer appreciated the extensive revision and the authors have addressed all concerns. The reviewer supports its publication at PLoS Computational Biology.

**Response**:

Thank you for your feedback. DGCyTOF utilizes calibration on the same experiment samples for assigning new cell types to their respective groups. In this study, our proposed model run CCA (Canonical Correlation Analysis) method to remove the batch effect prior to using this model.

**Reference**

[1] Butler A, Hoffman P, Smibert P, Papalexi E, Satija R. Integrating single-cell transcriptomic data across different conditions, technologies, and species. Nat Biotechnol. 2018, 36:411–20.

[2] Hardoon DR, Szedmak S, Shawe-Taylor J. Canonical correlation analysis: an overview with application to learning methods. Neural Comput. 2004, 16:2639–64.

[3] Yongsheng Ding, Lijun Cheng, Witold Pedrycz, Kuangrong Hao. Global nonlinear kernel prediction for large data set with a particle swarm-optimized interval support vector regression. IEEE transactions on neural networks and learning systems, 2015, 10(26):2521-2534.
